# Caffeic acid hinders the proliferation and migration through inhibition of IL-6 mediated JAK-STAT-3 signaling axis in human prostate cancer

**DOI:** 10.32604/or.2024.048007

**Published:** 2024-11-13

**Authors:** YUAN YIN, ZHENGYIN WANG, YUJIE HU, JIA WANG, YI WANG, QUN LU

**Affiliations:** 1 Department of Laboratory Medicine, Shanghai Traditional Chinese Medicine-Integrated Hospital, Shanghai University of Traditional Chinese Medicine, Shanghai, 200082, China; 2 Department of Acupuncture and Moxibustion, Shanghai Traditional Chinese Medicine-Integrated Hospital, Shanghai University of Traditional Chinese Medicine, Shanghai, 200082, China

**Keywords:** Caffeic acid (CA), Signal transducer and activating transcription-3 (STAT-3), Prostate cancer, Proliferation, Apoptosis

## Abstract

**Background:**

Caffeic acid (CA) is considered a promising phytochemical that has inhibited numerous cancer cell proliferation. Therefore, it is gaining increasing attention due to its safe and pharmacological applications. In this study, we investigated the role of CA in inhibiting the Interleukin-6 (IL-6)/Janus kinase (JAK)/Signal transducer and activator of transcription-3 (STAT-3) mediated suppression of the proliferation signaling in human prostate cancer cells.

**Materials and Methods:**

The role of CA in proliferation and colony formation abilities was studied using 3-[4,5-dimethylthiazol-2-yl]-2,5 diphenyl tetrazolium bromide (MTT) assay and colony formation assays. Tumour cell death and cell cycle arrest were identified using flow cytometry techniques. CA treatment-associated protein expression of mitogen-activated protein kinase (MAPK) families, IL-6/JAK/STAT-3, proliferation, and apoptosis protein expressions in PC-3 and LNCaP cell lines were measured using Western blot investigation.

**Results:**

We have obtained that treatment with CA inhibits prostate cancer cells (PC-3 and LNCaP) proliferation and induces reactive oxygen species (ROS), cell cycle arrest, and apoptosis cell death in a concentration-dependent manner. Moreover, CA treatment alleviates the expression phosphorylated form of MAPK families, i.e., extracellular signal-regulated kinase 1 (ERK1), c-Jun N-terminal kinase (JNK), and p38 in PC-3 cells. IL-6 mediated JAK/STAT3 expressions regulate the proliferation and antiapoptosis that leads to prostate cancer metastasis and migration. Therefore, to mitigate the expression of IL-6/JAK/STAT-3 is considered an important target for the treatment of prostate cancer. In this study, we have observed that CA inhibits the expression of IL-6, JAK1, and phosphorylated STAT-3 in both PC-3 and LNCaP cells. Due to the inhibitory effect of IL-6/JAK/STAT-3, it resulted in decreased expression of cyclin-D1, cyclin-D2, and CDK1 in both PC-3 cells. In addition, CA induces apoptosis by enhancing the expression of Bax and caspase-3; and decreased expression of Bcl-2 in prostate cancer cells.

**Conclusions:**

Thus, CA might act as a therapeutical application against prostate cancer by targeting the IL-6/JAK/STAT3 signaling axis.

## Introduction

Prostate cancer (PC) is thought to be the second most significant kind of cancer that causes death in men worldwide [1]. In 2023, it was estimated that 288,300 men in the United States were diagnosed with prostate cancer. Globally, the year 2020 saw an estimated 1,414,259 people diagnosed with prostate cancer. It is the fourth most commonly diagnosed cancer in the world [2]. PC has significantly increased daily in China due to several factors such as lifestyle changes, life expectancy in men, race, age, and heredity [3]. Unfortunately, the incidence of PC has been expected to enhance in the next 20 years [4]. Generally, chemotherapy-associated treatment strategies were used to inhibit the proliferation of prostate cancer. However, it produces serious adverse toxicities that were observed in the patients [5]. Hence, identifying pathological mechanisms underlying PC proliferation and metastasis is crucial for preventive and therapeutic strategies.

Mitogen activating protein kinases (MAPKs) families, including c-Jun N terminal kinases (JNK), extracellular signal-regulated kinase-1 (ERK1), and MAPK p38 highly expressed in prostate cancer that leads to activate numerous factors resulting in proliferation and invasion [6]. The scientific evidence has documented that proteins ERK1/2 regulated PC growth and differentiation. Also, JNK and p38 involve enhancing the regulation of inflammation, proliferation, apoptosis, etc. [7,8]. MAPK proteins highly activate numerous transcription factors that result in subsequent cancer cell proliferation [9]. Signal transducer and activator of transcription-3 (STAT-3), a crucial transcriptional factor that controls the expression of genes related to metastasis, inflammation, angiogenesis, cell survival, and proliferation [10]. Aberrant activation of STAT-3 translocation into the nucleus enhanced proliferation, cell cycle, and anti-apoptosis [11]. Interleukin-6 (IL-6) arbitrates inflammation while concomitantly regulating MAPK and JAK/STAT oncogenic pathways. STAT3 is a primary downstream regulator of IL-6 expression with its distinctive role in adaptable proliferation and neoplastic transformation in prostate cancer [12,13]. Therefore, inhibiting or inactivating IL-6 associated JAK-STAT-3 expression is thought to be a novel target for preventing PC proliferation and metastasis.

Damaged cells are prevented from replicating and multiplying due to a natural process known as cell cycle arrest. It has been controlled by the cyclin B1-cyclin-dependent kinase-1 (CDK1) complex. Blocking this complex with the p21 inhibitor causes a cell cycle arrest at the G2 checkpoint [14]. Damaged or improperly replicated DNA can be repaired during this pause in the cell cycle [15]. Numerous traditional chemotherapies were used for the first-line treatment of PC. However, it can also harm healthy cells, have unpleasant side effects on the patients, and affect normal cell growth [16]. Therefore, phytochemicals are considered a suitable replacement because they have multiple signaling and are less toxic than conventional chemotherapy [17].

Caffeic acid (CA), a type of polyphenolic compound, is derived from natural sources such as olives, berries, potatoes, and carrots. However, it is most abundantly found in coffee beans [18]. CA is additionally a component of hydroxycinnamic acid, a frequently encountered dietary ingredient in the daily consumption of humans [19]. It has shown plenty of pharmacological roles such as antioxidants, anticancer and antidiabetic, etc. [19,20]. Caffeic acid phenyl ester (CAPE), inhibits prostate cancer cell migration by suppressing EGFR/FAK/Akt signaling [21]. Previously CA impedes the expression of STAT3, thereby inducing apoptosis in UVB-exposed skin cancer models [22]. CA and its derivative CAPE also inhibit prostate cancer cells through numerous signaling events [23–25]. However, CA treatment on IL-6 mediated JAK-STAT-3 signaling axis in PC has not yet been studied. Therefore, in this study, we investigated the role of caffeic acid impedes the proliferation and migration of prostate cancer by inhibiting IL-6 mediated JAK-STAT-3 in prostate cancer cells.

## Materials and Method

### Reagents and chemicals

All the cell culture components such as Dulbecco’s modified eagles medium (DMEM), penicillin-streptomycin mixture, 0.25% trypsin-EDTA, phosphate-buffered saline (PBS), and fetal bovine serum (FBS) were obtained from Invitrogen Life Technologies (Carlsbad, CA, USA). Caffeic acid, 2,7-diacetyl dichlorofluorescein (DCFH-DA), 3-(4, 5-dimethylthiazol-2-yl), 2, 5-diphenyl tetrazolium bromide (MTT), crystal violet, ethidium bromide (EtBr), RIPA buffer, and acridine orange (AO) was purchased from Sigma (St. Louis, MO, USA). The primary anti-mouse and anti-rabbit monoclonal antibodies specific for cyclin D1 and D2, cyclin-dependent kinase 6 (CDK-6), interleukin 6 (IL-6), Janus kinase 1 (JAK-1), Extracellular signal-regulated kinase 1 (ERK-1), p38, p-STAT-3 (tyr705), caspase-3, Bcl-2, Bax, and β-actin and secondary antibodies were obtained from cell signaling technology, USA. The work required the use of analytical and molecular grades of fine chemicals and solvents.

### Cell culture

The human prostate cancer cells such as PC-3 and LNCaP were bought from the Chinese Academy of Sciences, Shanghai, China. DMEM medium was supplemented with 10% FBS and maintained at 37°C in a humidified environment containing 5% CO_2_ and 95% air environment, which were used to grow and culture the cells. The both cell lines were routinely screened for *Mycoplasma* contamination using the PCR-based Mycoplasma Detection Kit (Clontech Laboratories). Cells dividing exponentially were treated with different concentrations of CA (5, 10, and 15 µM) for 24 h.

### MTT assay

To assess the cytotoxicity potential of CA treatment against PC-3 and LNCaP cells by MTT assay [26]. In microtiter plates with a final volume of 100 *µ*L and MEM medium, cells were seeded at a density of 5000–10,000 cells per well. The plates were then incubated for 24 h. Cells were exposed to different concentrations of CA (0.39–50 µM) and allowed to react for 24 and 48 h, respectively, before 100 *µ*g of the MTT solution was added to each well and incubated for 4 h at 37°C. The purple formazan was dissolved using 100 *µ*L of DMSO after removing the MTT reagent. In an ELISA plate reader, the plate was read at 570 nm.

### Assay for colony formation

Prostate cancer cells (PC-3 and LNCaP) were seeded uniformly, added at the range of 5000 cells, and incubated for 24 h. Following that, CA was added to the cell culture in doses (of 5, 10, and 15 µM) and allowed to grow for 7 days in the CO_2_ incubator. The resulting colonies were washed with PBS and fixed with 3:1 glacial acetic acid and methanol. The cells were stained with 0.5% crystal violet for 20 min, and images were captured using a digital camera.

### ROS measurement

DCFH-DA was used to carry out the assay for cellular ROS detection [27]. In 6-well cell culture plates, cells were seeded (5 × 10^5^ cells/well) and incubated for the night. The cells were then given doses of CA (5, 10, and 15 µM) for 24 h. After that, the cells were exposed to 10 µM DCFDA for 30 min at 37°C. The cells were then removed and cleaned with DPBS, and their ROS content was determined using a FACS Calibur flow cytometer.

### Analysis of cell cycle

The cells were seeded in separate Petri dishes and treated with CA (5, 10, & 15 µM) for 24 h. After cells were harvested with Trypsin-EDTA and washed with PBS. They were plated in 15 ml conical tubes containing growth medium. After collecting the cells, they were fixed in 70% ethanol for 30 min after being centrifuged at 300 g for 10 min at 4°C. After being cleaned with PBS, the fixed cells were stained with the PI cocktail (50 g/ml PI and 50 g/ml RNase) for 30 min at room temperature (20°C–25°C) in the dark. The cell cycle was analyzed using a FACS Calibur flow cytometer (BD Biosciences; Franklin Lakes, NJ, USA).

### Apoptosis assay

In a 6-well cell culture plate, PC-3 cells (5 × 10^5^ cells/well) were seeded before being exposed to CA at various concentrations (5, 10, and 15 µM). The cells were removed after 24 h, rinsed twice with PBS, and stained for 15 min in the dark at room temperature (20°C–25°C) using FITC, Annexin V, and 7-AAD. The FACS Calibur flow cytometer was used to gauge the level of apoptosis.

### Western blot analysis

The CA treatment-mediated prostate cancer cells (PC-3 and LNCaP) were lysed in RIPA buffer for 30 min at 4°C, after which the lysate was centrifuged at 15000 g for 20 min at 4°C to extract the protein [28]. The obtained supernatant’s total protein concentration was utilized by the Bradford protein assay to determine (Bio-Rad, Hercules, CA, USA). Proteins were subjected to electrophoretic separation after being loaded in triplicate onto SDS-PAGE (10%–12%) gels and then transferred to a nitrocellulose membrane. The membranes were incubated with appropriate primary antibodies with dilutions of 1:1000 at 4°C overnight after blocking for 30 min in TBS-T (0.1%) Tween 20 solution containing 5% skim milk. The membranes were cleaned twice in TBS-T solution before being treated for an hour at room temperature with an HRP-linked secondary antibody with dilution of 1:2000. Chemiluminescence detection was done using Immobilon western chemiluminescent HRP substrate (Merck Millipore, Burlington, MA, USA).

### Statistical analysis

The values are expressed as mean ± SD values and were calculated using Graphpad Prism 5.0 (Graphpad Software Inc., San Diego, CA, USA). To identify the statistically significant differences between groups, one-way ANOVA was used. Statistics were considered significant at *p* < 0.05. All the experiments were used in triplicates (N = 3).

## Results

### CA induces cell death and inhibits colony formation in prostate cancer cells

**Figure 1:**
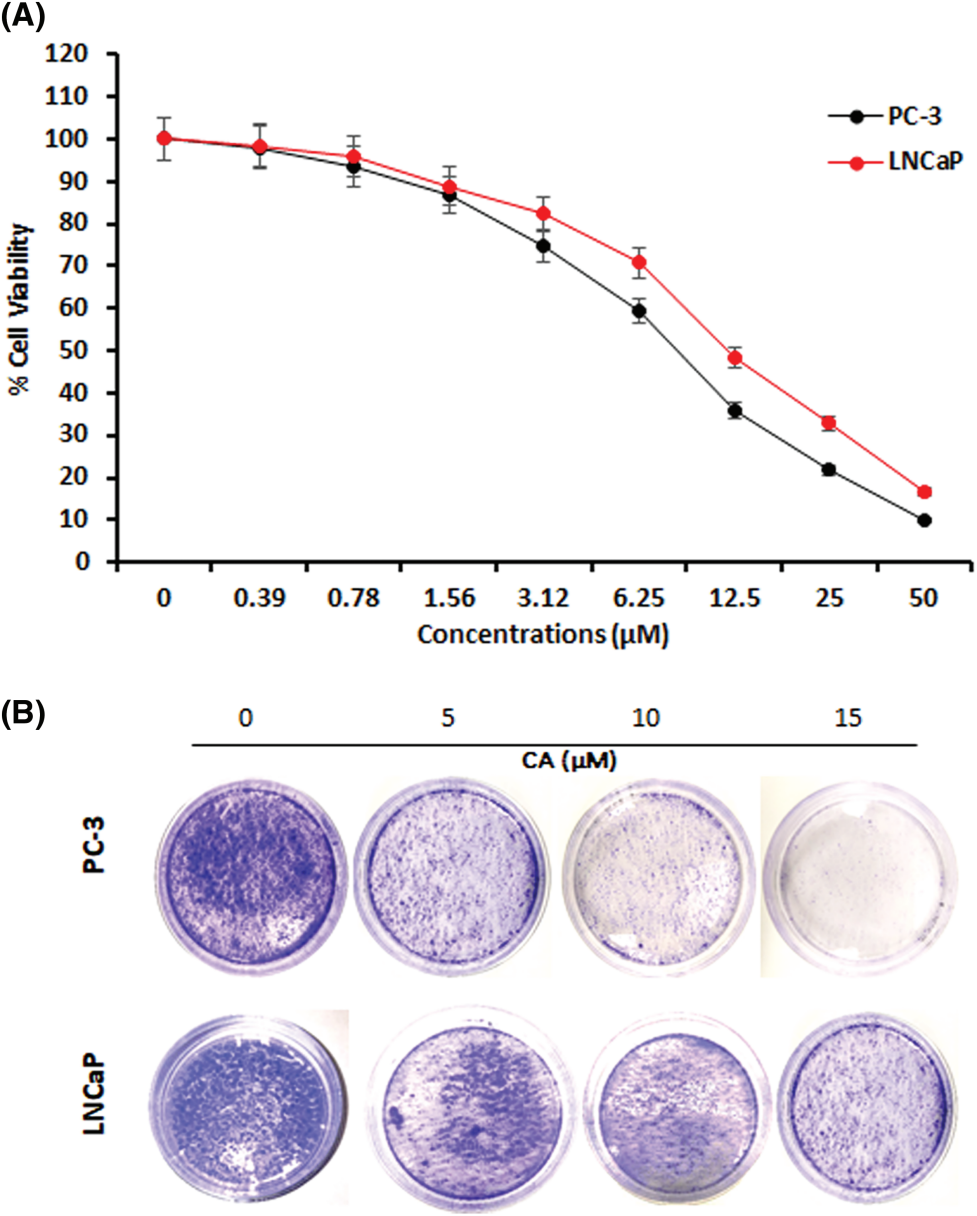
The effect of CA on cell viability and colony formation in prostate cancer cells. (A) Cytotoxicity potential of CA on PC-3 and LNCaP cells. Statistical analysis revealed a significant difference (*p* < 0.05) in cell viability between the CA-treated group and the control group, indicating the cytotoxic effects of CA on the prostate cancer cells. (B) CA treatment-mediated colony formation was studied by crystal violet staining.

This study determines the cytotoxicity of CA treatment against prostate cancer cells such as PC-3 and LNCaP viability and colony formation. Fig. 1A shows that CA treatment significantly reduced PC-3 and LNCaP cell viability in concentration dependency. The increasing concentration of CA (0.38–50 µM) enhances the toxicity of PC-3 and LNCaP cells. The inhibitory concentration of 50% (IC_50_) of CA was 9.0 µM for PC-3 cells and 11.5 µM for LNCaP cells. Also, we observed that CA (25–50 µM) is more toxic in PC-3 cells and LNCaP cells. Hence, we selected 5, 10, and 15 µM concentrations of CA for further studies. Moreover, the role of CA treatment associated with colony formation in prostate cancer cells such as PC-3 and LNCaP cells was examined by the crystal violet assay. As Fig. 1B shows, PC-3 and LNCaP cells were exposed to different concentrations of CA and consequently incubated at 37°C for 14 days. We have obtained that CA can impede the formation of colonies in PC-3 cells. CA 15 μM concentration has been observed to inhibit colonies in both PC-3 and LNCaP cells.

### CA induces ROS production, G2/M cell cycle arrest and apoptotic cell death in prostate cancer cells

**Figure 2:**
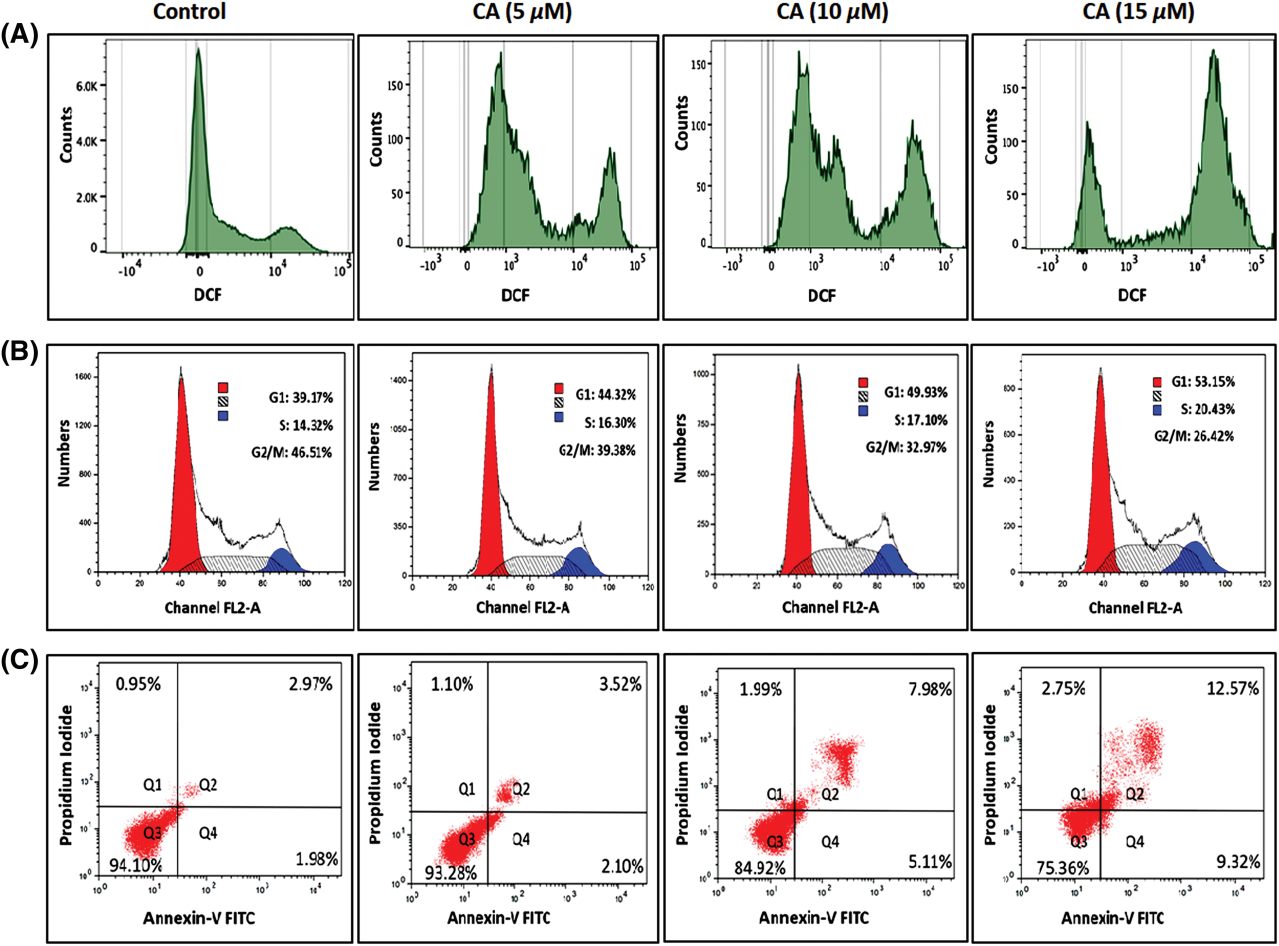
CA enhances reactive oxygen species generation, cell cycle arrest and apoptotic cell death in PC-3 cells. (A) Cells were treated with varying concentrations of CA for a duration of 24 h. Subsequently, they were exposed to DCFH-DA, and ROS was quantified by flow cytometry. (B) CA treatment mediated different phases of the cell cycle was assessed by flow cytometer. Histograms represented the percentages of different cell cycle phases such as G1, S, and G2/M Populations. (C) The role of CA treatment associated with apoptosis of PC-3 cells stained with Annexin V and PI and quantified by flow cytometer. Histograms represented the percentages of apoptosis and viable populations.

CA treatment on overproduction of ROS in PC-3 cells was studied by DCFH-DA staining. CA treatment with PC-3 cells shows a significantly increased amount of DCF fluorescence, and the histogram indicates the production of ROS in fluorescence intensity (Fig. 2A). The maximum fluorescence was observed in CA (15 µM) when comparing other concentrations in PC-3 cells.

Moreover, the role of CA treatment associated with different phases of cell cycle arrest in PC-3 cells was examined by flow cytometry. Fig. 2B found that CA treatment prompted their build-up at the G1 cell cycle phase in PC-3 cells. Also, CA treatment enhances the G1 phase percentage from 44.32% to 53.25% in a concentration-dependent manner. Moreover, we observed that CA treatment decreased the G2/M Phase in PC-3 cells. This could indicate that CA arrests the cell cycle at the phase of G2/M.

In addition, the role of CA treatment associated with apoptosis in PC-3 cells was quantified by Annexin V and PI staining (Fig. 2C). To perceive the apoptotic potential of CA treatment for 24 h showed an induction of apoptosis in PC-3 cells. Also, we observed that CA treatment increased the percentages of apoptotic cells in PC-3 cells.

### CA inhibited the phosphorylation of MAPK proteins in prostate cancer cells

**Figure 3:**
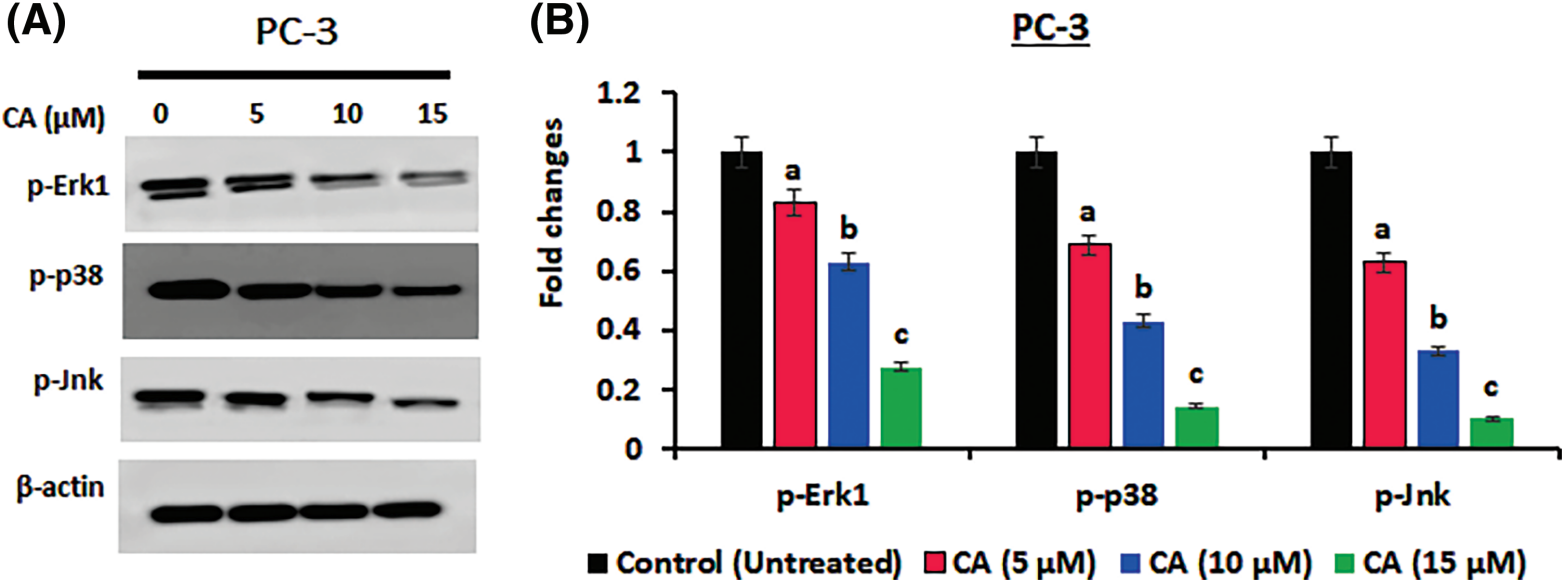
CA suppresses phosphorylation of MAPK proteins in prostate cancers. (A) Western blotting images were used to visualize the phosphorylation levels of p38, JNK, and ERK-1 expressions in PC-3 cells. (B) The bar diagram exhibits the mean ± SD of the relative gene expression obtained from three independent experiments. The results show significant differences compared to the control protein β-actin, with a statistical significance level of *p* < 0.05. Data (a, b, c) are statistically significant when comparing with other groups.

The JNK, p38, and Erk1 signaling pathways are essential for oxidative stress. In the current study, JNK, p38, and ERK1 protein phosphorylation in prostate cancer cells PC-3 were examined by western blotting. As Fig. 3 shows, the overexpression of phosphorylated JNK, p38, and Erk1 was observed in untreated PC-3 cells. CA treatment with PC-3 cells appreciably reduced the expression of the phosphorylated form of JNK, p38, and Erk1. These results imply that CA treatment hinders oxidative stress.

### CA inhibited IL-6 mediated JAK-STAT-3 expression in the prostate cancer cells

**Figure 4:**
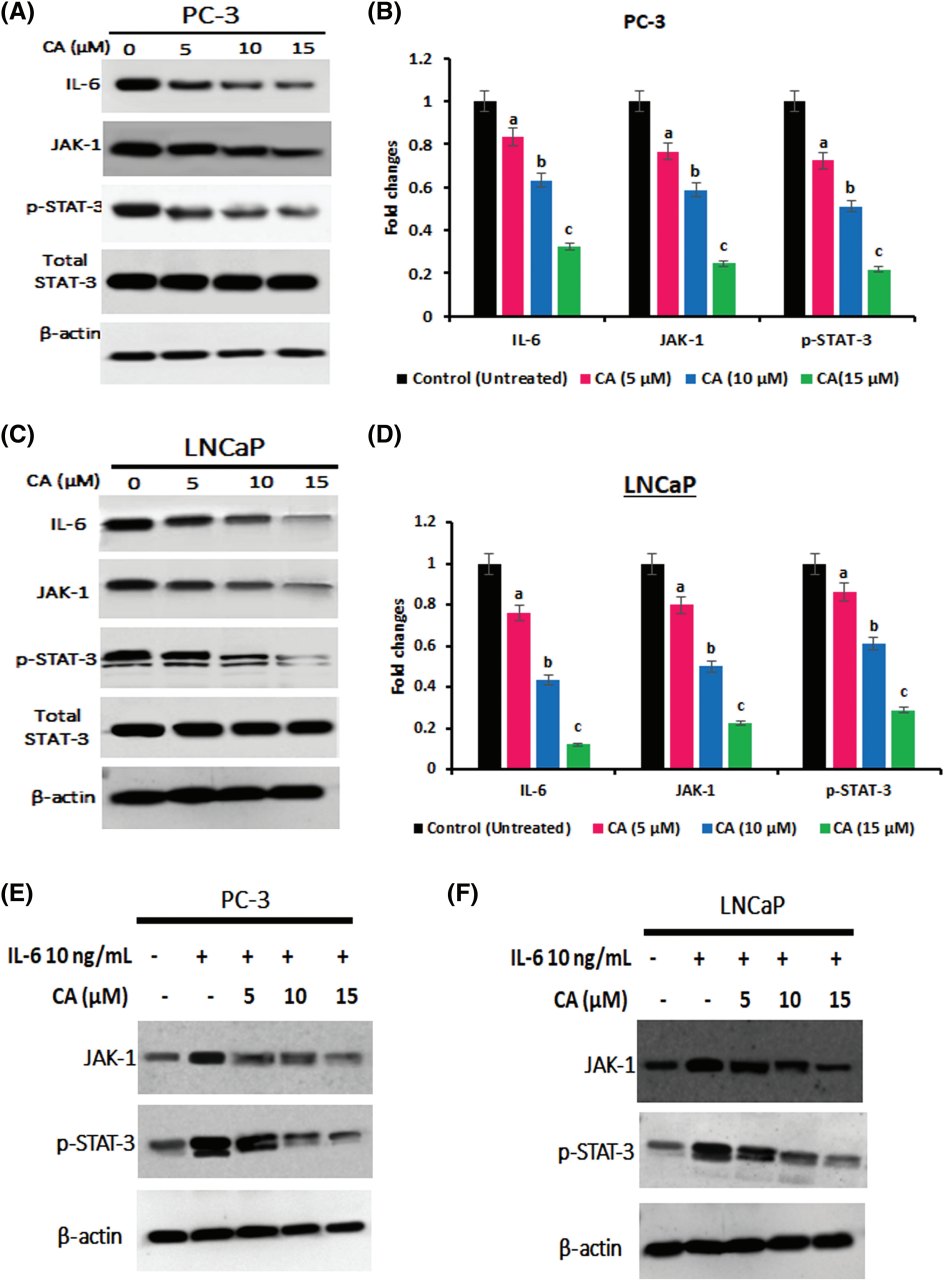
CA attenuates IL-6/JAK/STAT signaling in prostate cancer cell line. (A) Western blotting images were obtained for IL-6, JAK-1, and p-STAT-3 (tyr705) proteins in PC-3 cells. (B) The bar diagram illustrates significant differences in relative protein expression compared to the control protein β-actin. Data has shown to mean ± standard deviation and data (a, b, c) are statistically significant comparing with other groups. (C) Western blotting images were obtained for IL-6, JAK-1, and p-STAT-3 (tyr705) proteins in LNCaP cells. (D) The bar diagram illustrates significant differences in relative protein expression compared to the control protein β-actin. Results indicated by the mean ± SD derived from three independent experimental assays (*p* < 0.05). Data (a, b, c) are statistically significant when comparing with other groups. (E and F) Western blot data on CA treatment on IL-6 induced expression of, JAK-1, and p-STAT-3 (tyr705) in PC-3 and LNCaP cells.

STAT3 is a crucial transcriptional gene stimulated by specific upstream elements such as ROS, interleukins, and tyrosine kinases that can regulate adverse roles to numerous proliferation and oncogenic proteins. CA treatment on IL-6, JAK1, and p-STAT-3 (tyr705) expression in prostate cancer cells PC-3 were examined by western blotting. As Figs. 4A–4D show, IL-6, JAK1, and p-STAT-3 (tyr705) overexpression were observed in untreated PC-3 and LNCaP cells. CA treatment with PC-3 and LNCaP cells appreciably reduced the expression of IL-6, JAK1, and p-STAT-3 (tyr705). These results imply that CA treatment impedes the IL-6/JAK/STAT-3 expression, thereby inhibiting proliferation and oxidative stress in PC-3 and LNCaP cells. In addition, CA treatment whether directly inhibits STAT-3 expressions was evaluated by IL-6 induced expression of JAK, and p-STAT-3 was evaluated by western blot. Figs. 4E and 4F show IL-6 treatment alone enhanced the expression of JAK and p-STAT-3 in prostate cancer cells. However, PC-3 and LNCaP cells were exposed to IL-6 following that CA treatment significantly reduced the expression of JAK-1 and p-STAT3 expression. The findings suggest that CA indirectly inhibits JAK-STAT3 expression in prostate cancer cells by suppressing IL-6 expression.

### CA inhibited the proliferation and anti-apoptotic protein expression in PC-3 cells

**Figure 5:**
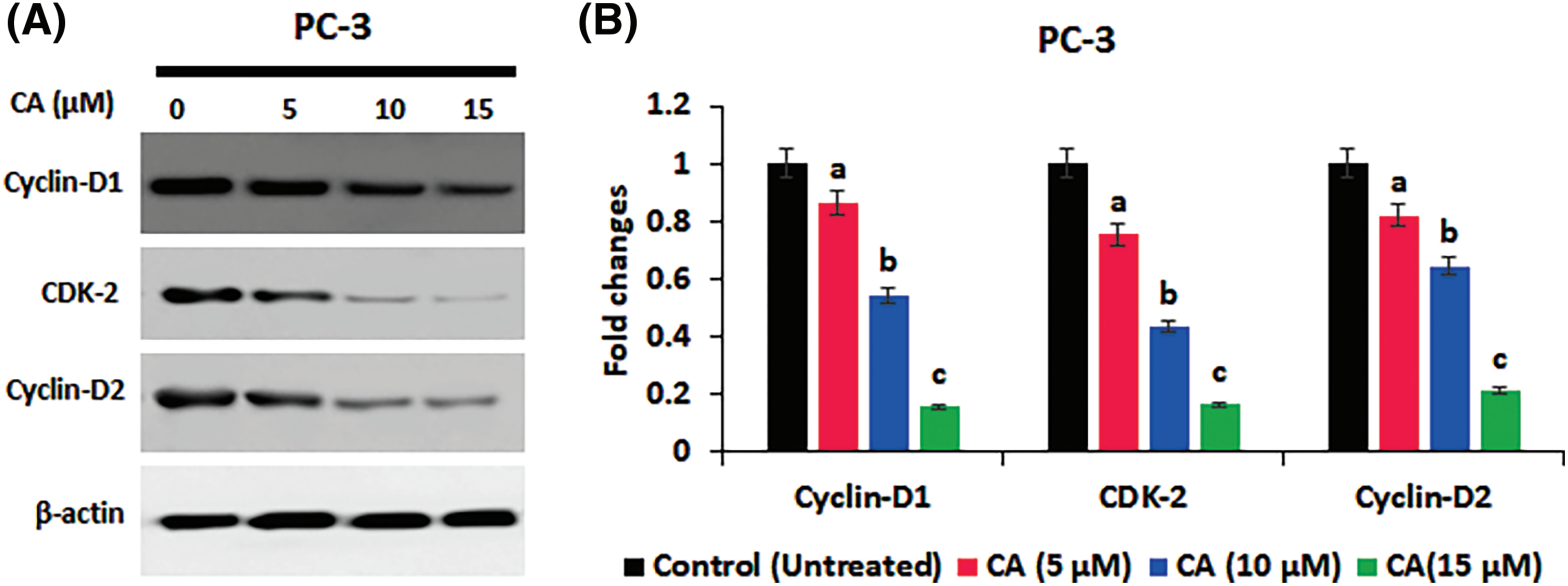
CA inhibited the proliferation marker expression in PC-3 cells. (A) Western blotting images were obtained for cyclin-D1, CDK-2, and cyclin-D2 protein expressions in PC-3 cells. (B) The bar diagram illustrates significant differences in relative protein expression compared to the control protein β-actin, as indicated by the mean ± SD derived from three independent experimental assays (*p* < 0.05). Data (a, b, c) are statistically significant when comparing with other groups.

**Figure 6:**
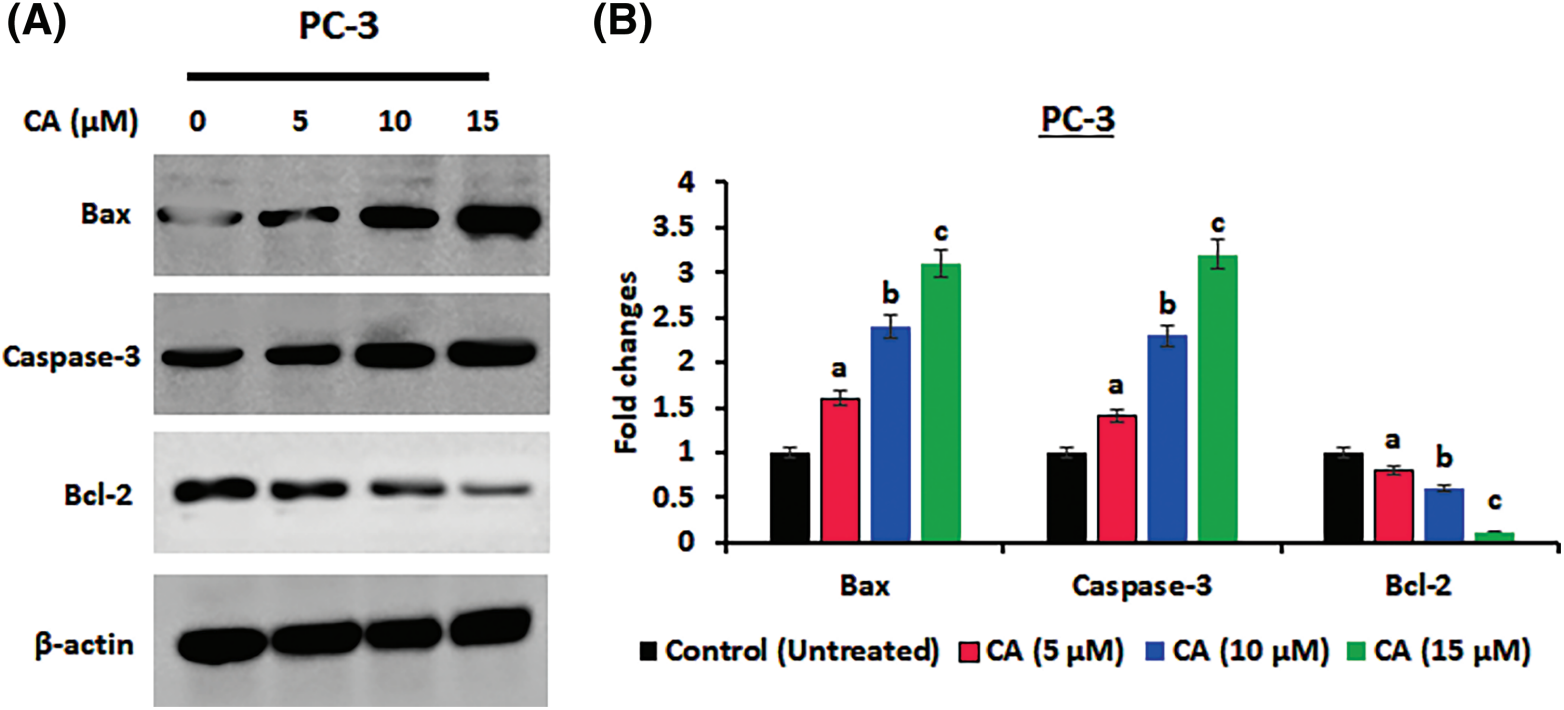
CA treatment enhances apoptosis protein expressions in PC-3 cells. (A) Western blotting images were obtained for Bax, Caspase-3, and Bcl-2 protein expressions in PC-3 cells. (B) The bar diagram illustrates significant differences in relative protein expression compared to the control protein β-actin, as indicated by the mean ± SD derived from three independent experimental assays (*p* < 0.05). Data (a, b, c) are statistically significant when comparing with other groups.

The role of CA treatment on proliferative proteins (Cyclin-D1, CDK-2, Cyclin-D2) expression in prostate cancer cells PC-3 was examined by western blotting. As Fig. 5 shows, the overexpression of proliferative proteins (Cyclin-D1, CDK-2, and Cyclin-D2) was observed in untreated PC-3 cells. CA treatment with PC-3 cells appreciably reduced the expression of proliferative proteins (Cyclin-D1, CDK-2, Cyclin-D2). In addition, CA treatment on apoptotic proteins (Bcl-2, Bax, and Caspase-3) expression in prostate cancer cells was examined by western blotting (Fig. 6). The overexpression of proapoptotic factor Bax and caspases; reduced the expression of anti-apoptotic factor Bcl-2 was observed in untreated control cells. However, CA treatment reverted the expression in PC-3 cells. These results imply that CA treatment obstructs the proliferation and induces subsequent apoptosis in PC-3 cells.

## Discussion

In this study, we examined the molecular mechanisms underlying the anti-cancer effects of caffeic acid (CA), a naturally occurring flavonoid, on prostate cancer cells. We observed that CA could block the IL-6 mediated STAT-3 signaling pathway, leading to apoptosis in prostate cancer cells. Prostate cancer is considered the deadliest cancer with a poor prognosis, particularly in men worldwide [29]. STAT-3 is frequently activated in prostate cancer, promoting tumor survival, growth, angiogenesis, and metastasis [30]. Hence, STAT-3 overexpression is thought to be a crucial target for the identification of new therapeutical molecules against prostate cancer. CA belongs to a polyphenol drug that has significant anti-tumoral activities [20]. In this study, we demonstrated that CA could inhibit the cell viability and colony formation of prostate cancer cells in a dose-dependent manner. CA IC50 value was discovered to be 9.0 µM for PC-3 cells and 11.5 µM for LNCaP cells. Previously, hydrocinnamic acids such as CA and ferulic acid treatment induced toxicity in human breast cancer cells whereas not in normal cells [31,32]. According to this research, CA has strong anti-proliferative effects on PC-3 and LNCaP cancer cells. In response to DNA damage brought on by various agents such as ultraviolet light, alkylating substances, biphenyls, and ionizing radiation, cells go through apoptosis and cell cycle arrest [13]. Numerous phytochemicals have been found to encourage cell cycle arrest and apoptosis in cancer cells [33]. The high amount of intracellular ROS can cause oxidative stress, which harms proteins, lipids, and DNA [34]. Moreover, the overproduction of ROS induces different cell cycle arrests and apoptosis due to the mechanisms of pro-oxidants [35]. In this study, CA highly enhances the intracellular ROS in PC-3 cells. This ROS production induces cell cycle arrest and apoptosis in PC-3 cells. Previously, numerous phytochemicals enhanced the ROS cells through pro-oxidants, thereby inducing cell death in several cancer cells. In various cancer cells, CA has been shown to induce early and late apoptosis and stop the cell cycle at the G2/M phase [36,37]. Flow cytometry studies demonstrated that CA triggered G2/M phase arrest in PC-3 cells by increasing the percentage of cells in the G0/G1 and S phases and decreasing the number of cells in the G2/M phase.

The MAPK signaling cascade is considered a major conserved event that controls extracellular stimuli like stress, cytokines, and growth factors that result in oxidative stress [38]. Dysregulation of MAPK-associated downstream elements has been linked to many pathological conditions, including prostate cancer. MAPK families are needed to activate STAT-3 phosphorylation, which is crucial for cell proliferation and apoptosis [9]. According to the results of our study, CA inhibited the phosphorylation of the MAPK protein, which may be related to its effect on the ERK pathway. Our western blot analysis showed that the levels of phosphorylated JNK, p38, and MAPK (ERK-1) protein were significantly reduced in CA-treated PC-3 cells. This result is compared with earlier research that found CA inhibits the ERK pathway’s activation in solar radiation-exposed skin cancer models [23].

Cyclins D1 and cyclin-D2 are proliferative markers that coordinate the cyclin-dependent kinase 4 and 6 (CDK4/CDK6) to control cell cycle conversion from G1 to the S phase [14]. The overexpression or activation of these proteins resulted in growth factor stimulation in human cancers [39]. In this current study, we have observed that CA downregulated Cyclin-D1, CDK-2, and Cyclin-D2 in PC-3 cells. Previously, natural remedies from pericarp extract of Baneh enhance G1 phase cell cycle arrest and downregulate the expression CDK and cyclin D1, thereby inhibiting human breast cancer [40]. Apoptosis was also influenced by CA, which was observed in upregulated pro-apoptotic proteins such as Bax and caspase-3 and downregulated anti-apoptotic protein Bcl-2 in PC-3 cells. These results align with earlier research that suggested natural substances can trigger apoptosis in cancer cells by regulating the expression of pro- and anti-apoptotic proteins [41].

STAT-3 over expressions associated with proliferative markers are highly observed in prostate cancer models [30]. Tyrosine kinases, interleukins, and interferons are upstream positive genes that activate the oncogenic transcription factor STAT3 [42]. When the activation of STAT3 in the cytosol, is translocated into the nucleus and enhances the transcriptional regulation, severe antiapoptotic and proliferation markers [11]. The overexpression of IL-6 and JAK-1 is required for the STAT-3 activations that lead to prostate cancer proliferation and migrations [13]. We noticed that the control cells had high p-STAT-3 in PC-3 and LNCaP cells. However, cells exposed to CA treatment markedly reduced the protein expression of IL-6 mediated JAK, p-STAT-3 and resulted in translocation of STAT-3 inhibition. To further verify whether CA directly inhibits STAT-3 expression, we investigated the CA on IL-6-induced expression of JAK and p-STAT-3. Our results confirm that exposure of PC-3 and LNCaP cells to IL-6, followed by CA treatment, significantly decreased the expression of JAK-1 and p-STAT3. These findings imply that CA indirectly hinders JAK-STAT3 expression in prostate cancer cells by suppressing IL-6 expression. Previously, carnosol, a phytonutrient from rosemary, impedes human colon cancer by suppressing the phosphorylation of STAT-3 [43]. Moreover, isoliquiritigenin inhibited the JAK2 and STAT-3-associated apoptosis in human renal carcinoma [44]. Based on this report, we have noticed that CA impedes the proliferation and migration of prostate cancer cells by inhibiting IL-6 mediated JAK-STAT3 signaling. This study primarily focused on the in vitro effects of CA on prostate cancer cells. However, its limitations include the lack of in vivo validation, which is necessary to confirm the CA therapeutic potential. Additionally, the dosage and bioavailability of CA have not yet studied. These factors represent critical limitations that need to be addressed in future studies.

## Conclusion

In this study, we concluded that CA exerts a significant antiproliferation nature against prostate cancer cells. We have observed that CA inhibits cell proliferation by enhancing cytotoxicity, cell cycle arrest, and apoptosis. Moreover, CA suppresses the proliferative markers, and MAPK proteins, and induces apoptosis through inhibiting the IL-6 mediated phosphorylation of JAK/STAT-3 expression in prostate cancer. Thus, caffeic acid might be a therapeutic application for prostate cancer, and *in vivo*, experimental models are needed for further evaluation.

## Data Availability

Data will be made available upon reasonable response.
